# Moral Injury and Pre-Deployment Personality Factors as Contributors to Psychiatric Symptomatology among Combatants: A Two-Year Prospective Study

**DOI:** 10.1192/j.eurpsy.2023.1307

**Published:** 2023-07-19

**Authors:** Y. Levi-Belz, G. Zerach

**Affiliations:** Ruppin Academic Cener, Emek hefer; 2 Ariel University, Ariel, Israel

## Abstract

**Introduction:**

Combatants who are exposed to events which transgress deeply held moral beliefs might face lasting psychopathological outcomes, referred to as Moral Injury (MI). However, knowledge about pre-deployment factors which might moderate the negative consequences of MI is sparse.

**Objectives:**

In this prospective study, we examined pre-enlistment characteristics and pre-deployment personality factors as possible moderators in the link between exposure to potentially morally injurious events (PMIEs) and psychiatric symptomatology among Israeli active-duty combatants.

**Methods:**

A sample of 335 active-duty Israeli combatants participated in a 2.5-year prospective study with three waves of measurements (T1: 12 months before enlistment, T2: 6 months following enlistment- pre deployment, and T3: 18 months following enlistment- post deployment). Participants’ characteristics were assessed via semi-structured interviews (T1) and validated self-report measures of personality factors: emotional regulation, impulsivity, and aggression (T2) and combat exposure, PMIEs, psychiatric symptomology and post traumatic symptoms (T3) between 2019-2021.

**Results:**

Pre-enlistment psychiatric difficulties and negative life events contributed to higher exposure to PMIEs post deployment. Higher levels of pre-deployment aggression and lower levels of emotional regulation and impulsivity moderated the association between betrayal, PMIEs and psychiatric symptomology post deployment, above and beyond pre-enlistment psychiatric difficulties and life events.

**Conclusions:**

Our results highlight that pre-deployment emotional regulation, impulsivity and aggressiveness levels should be assessed, screened, and identified among combatants, as they all facilitate psychiatric symptomology (and PTSS) after combatants are exposed to PMIEs of betrayal. Such pre-assessment will enable identification of at-risk combatants and might provide them with tailor made preparation regarding moral and ethical situations that should be investigated in future researches.

**Disclosure of Interest:**

Y. Levi-Belz: None Declared, G. Zerach Shareolder of: no, Grant / Research support from: no, Consultant of: no, Employee of: no, Paid Instructor of: no, Speakers bureau of: no

